# Monkeypox: A Mini-Review on the Globally Emerging Orthopoxvirus

**DOI:** 10.3390/ijerph192315684

**Published:** 2022-11-25

**Authors:** Amy Evans, Bara’ Abdallah AlShurman, Hibah Sehar, Zahid Ahmad Butt

**Affiliations:** School of Public Health Sciences, University of Waterloo, Waterloo, ON N2L 3G1, Canada

**Keywords:** monkeypox, orthopoxvirus, communicable diseases

## Abstract

Monkeypox is a zoonotic infectious disease belonging to the orthopoxvirus family that has predominantly occurred in West and Central Africa since it was initially discovered in 1958. In May 2022, a global outbreak of monkeypox began to occur on an international scale, with case numbers still rising as this review is being written. This mini review sought to analyze the existing literature on monkeypox published from 2017 onward to provide epidemiological context to current outbreaks. PubMed and Google Scholar databases were used to gather both peer-reviewed and grey literature on the routes of transmission, case definitions, clinical characteristics, diagnosis, management, prevention, vaccination, and epidemiology of monkeypox. Epidemiological studies indicate that the age of onset of monkeypox has increased over time. Antivirals, such as Tecovirimat and Brincidofovir, are recommended to manage confirmed cases of monkeypox. Although mass vaccination is not currently recommended, the smallpox vaccine can be used as a preventative measure for at-risk groups, such as men who have sex with men and frontline healthcare workers. Further peer-reviewed research addressing animal reservoirs and sexual transmission dynamics is needed.

## 1. Introduction

Monkeypox is a zoonotic virus belonging to the Poxviridae family, Chordopoxvirinae subfamily and genus Orthopoxvirus [1]. It was first discovered in Democratic Republic of the Congo in a 9-month-old infant male in 1970 and eventually became endemic in Central and West Africa [2]. It’s routes of transmission include direct contact with an infected individual, animal, or contaminated objects such as towels or clothing [3].

The number of confirmed and/or probable cases of the human monkeypox virus has substantially increased globally within the last five years [4]. Several studies have documented human monkeypox outbreaks occurring in both endemic and non-endemic countries from 2017 to present [5,6], with the majority of the literature describing an outbreak that occurred in Nigeria from 2017 to 2018 [7,8]. During this outbreak of the West African clade of the monkeypox virus, 122 probable or confirmed cases of monkeypox were witnessed in 17 Nigerian states [7]. Additionally, four cases of human monkeypox virus occurring throughout this outbreak were reported in Singapore (*n* = 1), Israel (*n* = 1), and the United Kingdom (*n* = 2) and were determined to have been transported to these non-endemic countries by individuals travelling from Nigeria during this time [8]. Epidemiological reports detailing the 2017–2018 monkeypox outbreak in Nigeria cite the need for further investigation into specific animal reservoirs responsible for transmitting the virus to humans and determining the extent of human-to-human transmission versus human-to-animal transmission [7,8].

In May 2022, the World Health Organization (WHO) began reporting significant increases in global monkeypox cases [9]. As of 20 October 2022, 75,166 laboratory confirmed cases of monkeypox across 109 countries have been reported [10]. On July 23, the WHO officially declared current global monkeypox outbreaks as a Public Health Emergency of International Concern (PHEIC) [11]. Interim guidance from the WHO advises that infection, prevention and control measures should be promptly administered to support the early detection of the virus, especially in at risk-communities [10]. However, peer-reviewed research investigating which communities are at the highest risk for contracting the monkeypox virus based on demographic and lifestyle factors is sparse [4].

Past reviews on the monkeypox virus largely centre around how the virus’s epidemiology has evolved over time [4,12,13]. Literature on current human monkeypox outbreaks occurring in non-endemic countries remains limited. As such, our purpose is to rapidly review the literature on monkeypox published from 2017 to 2022 to provide epidemiological context for current monkeypox outbreaks occurring in non-endemic countries and to provide recommendations for future research.

## 2. Materials and Methods

We conducted a rapid mini review to do narrative synthesis and review the epidemiological aspects that have been published about recent and past outbreaks of Monkeypox. Since there is no agreed methodology for conducting mini reviews, we followed a non-systematic search approach within a tight time frame. Therefore, only PubMed and Google Scholar were used to search for articles. The search strategy was done using indexing terms pertaining to “Monkeypox AND Adults AND Outbreak”. To reach more international data, we searched three sources of grey literature: the WHO and the European and American Centres for Disease Control and Prevention ((E)CDC). Official Canadian government websites were also accessed, such as Health Canada and the Public Health Agency of Canada, to find more information about Monkeypox cases in Canada. Our search was limited to English-language articles published between 2017 and 2022.

Regarding our broad inclusion and exclusion criteria, only articles that focused on adults > 18 years old were included. Relevant articles were identified as those related to human monkeypox, its epidemiologic and clinical characteristics, diagnosis, management, prevention, and re-emergence of outbreaks. Due to the limited number of primary research studies on monkeypox, non-peer reviewed papers (e.g., perspectives, opinions, and editorials) were included. Lastly, all countries were eligible to be included in this study.

## 3. Results

Our findings were organized into five categories: (1) Sources and routes of transmission, (2) Case definitions and clinical characteristics (3) Diagnosis/Management, (4) Prevention/Vaccination, and (5) Epidemiology.

### 3.1. Sources and Routes of Transmission

Modes of transmission regarding monkeypox include animal-to-human or human-to-human transmission [7,14]. The CDC has advised that coming into direct contact with vesiculopustular lesions, bodily fluids, and/or respiratory droplets from an infected animal or human during face-to-face and physical contact, as well as touching contaminated items, elevates the risk of transmission [7,15]. A case report in the UK also identified consuming infected bushmeat as a potential factor of transmission [5]. Although monkeypox is a zoonotic infectious disease, Falendysz et al., reported that animal reservoirs remain largely unknown [16]. However, some animal species that have previously infected humans with monkeypox in endemic regions include rope squirrels, pouched rats, and non-human primates in the DRC [16]. Risk factors and determinants of contracting monkeypox remain lesser known and are often context dependent [4,9,17]. A study conducted in the DRC that assessed risk indices associated with contracting monkeypox found that housing quality and occupation were associated with the risk of infection [17]. More specifically, individuals who reported residing in lower quality housing and/or who identified as a hunter or farmer (or as someone who performs hunting and farming activities) were at a greater risk of contracting monkeypox [17]. Additionally, the WHO suggested that smallpox vaccine status may also be related to the likelihood of contracting monkeypox, but more research is needed [9].

### 3.2. Case Defintions and Clinical Characteristics

Previous studies have reported three case definitions of monkeypox based on the patients’ symptoms as well as laboratory examinations [4,7]. A suspected case was described as an individual presenting with a sudden onset of fever followed by a maculopapular rash concentrated on the face, palms of the hands, and soles of the feet. A confirmed case was described as a suspected case with laboratory confirmation through methods such as PCR testing, isolation of the virus, and positive antibody detection. Lastly, a probable case was described as a suspected case unable to obtain laboratory confirmation but with an epidemiological link to a confirmed case.

Clinical symptoms of monkeypox include sudden onset fever, lymphadenopathy, and a rash consisting of vesiculopustular lesions throughout the body [7]. A recent review of monkeypox found that lesions typically occur 1–3 days after the onset of lymphadenopathy and fever [18], and cover the face, limbs, trunk, genital, and perianal areas [6]. Other symptoms reported by the CDC include backaches, muscle aches, and fatigue/exhaustion [15]. Additionally, a retrospective study in the UK reported low mood in some patients after acquiring monkeypox, but it was unclear whether this was due to the virus itself or a result of isolation [6]. According to Vaughan et al., monkeypox has an incubation period ranging from 5 to 21 days, and recovery usually occurs 14 to 21 days after onset [5]. Similarly, Beer and Rao found that individuals are considered infectious from the time of the rash onset until lesions dry up and the rash dissipates about 3 to 4 weeks later [13]. Another review published in 2018 reported that complications from monkeypox, such as sepsis and septic shock from elevated immune responses, as well as secondary bacterial infections from skin lesions, may occur after contracting the virus [12].

### 3.3. Diagnosis/Management

As listed by the WHO, rash illnesses, such as measles, smallpox, chickenpox, and syphilis are some of the differential diagnoses illnesses that must be ruled out to determine if the rash is characteristic of monkeypox [19]. During the prodromal phase, lymphadenopathy can be used to differentiate monkeypox from both smallpox and chickenpox [19]. In terms of accuracy and sensitivity, the recommended test for diagnosis is Polymerase chain reaction (PCR), using samples from skin lesions [19]. Samples include dry crust and fluid from the vesicles and pustules. According to the CDC, multiple samples from different regions of the body and different appearances are typically collected [20]. Additionally, PCR physicians should also be provided records of the patient history, including when the rash appeared, the onset date of fever, current patient status, and when the specimen was collected [19].

The management and treatment of monkeypox primarily involves symptom management [21]. Current CDC guidelines recommend antivirals such as Tecovirimat and Brincidofovir, as well as Vaccinia Immune Globulin, to manage monkeypox [22]. A review on the treatment of monkeypox stated that Tecovirimat stops the spread of the virus within the host by inhibiting the viral envelope protein, VP37 [22]. Thus, the virus cannot become fully mature and spread further. Rizk et al. reported that the efficacy of Tecovirimat has not been tested in humans for monkeypox, however against the placebo it has shown increased survival rates in animal studies [22]. Brincidofovir, another antiviral, was recently approved for smallpox treatment in June 2022 [22]. It is an analog of Cidofovir and similarly inhibits DNA polymerase but is less nephrotoxic [22]. According to Rizk et al., Brincidofovir has been shown to be effective against orthopoxvirus infections, but a lack of human trials remains to be conducted [22]. Liver tests must be performed before and during the administration of Brincidofovir to prevent potential adverse events, such as increased serum transaminases and serum bilirubin, from occurring [22]. Finally, Vaccinia Immune Globulin (VIG) was suggested to be used to treat complications arising from the use of vaccinia vaccines to prevent monkeypox [22]. The most common reported complications are vaccinatum, severe generalized vaccinia, progressive vaccinia, and secondary infections [22].

### 3.4. Prevention/Vaccination

While the current threat to the general population is low, methods to prevent the spread of monkeypox are consistent with methods used to prevent viruses in general [23]. The CDC recommends limiting contact with infected or potentially infected animals and/or humans and avoiding contact with objects that may have had contact with infected animals and/or humans, such as towels, linen, and utensils [23]. Quarantining infected individuals, practicing hand hygiene, and using alcohol-based sanitizers are also recommended to help protect against contracting monkeypox [19,23]. For healthcare workers, using personal protective equipment when interacting with patients or infected individuals decreases the risk of transmission [24]. Additionally, as stated by the GC, individuals who have a suspected or confirmed case of monkeypox, as well as anyone they interact with, should wear a mask when interacting with others [25].

The World Health Organization does not currently recommend vaccination against monkeypox in the general population; however, it does recommend vaccination for at-risk groups, such as men who have sex with men (MSM), healthcare workers, and laboratory technicians [26]. The Advisory Committee on Immunization Practices (ACIP) has recommended two vaccines previously developed for pre-exposure prophylaxis against smallpox in the prevention of monkeypox: Jynneos™ and ACAM2000 [27]. Jynneos™ is a live, attenuated vaccine that was approved by the Food and Drug Administration (FDA) in 2019 [27]. It was originally created from the replication-deficient modified vaccinia Ankara-Bavarian Nordic (MVA-BN strain) [22]. ACIP reports that Jynneos™ can be used by individuals who are 18 or older as a 2-dose series with 28 days between the first and second injection [27]. Additionally, while ACIP also reports no notable adverse events from the vaccine, they advise that more research is still needed regarding the effectiveness of a single dose, as well as guidance in administering Jynneos™ alongside COVID-19 vaccines [27].

According to Rizk et al., a second vaccine, ACAM2000, was originally approved for persons who are at high risk for smallpox in 2007 [22]. Like Jynneos™, it is a live replication-competent vaccinia virus and is administered percutaneously as a single dose using a bifurcated needle [22]. Currently in the US, it is under investigational new drug (IND) protocol and can be used for monkeypox should there be an outbreak [22]. Rizk et al., report that unlike Jynneos™, ACAM2000 does create a cutaneous reaction at the inoculation site and the risk of inadvertent inoculation or autoinoculation exists [22]. They also include in their review that individuals with atopic dermatitis or eczema can experience eczema vaccinatum and that individuals who are immunocompromised can experience progressive vaccinia after using ACAM2000 [22]. Thus, ACAM2000 is not recommended to be used among populations with high numbers of immunosuppressed persons [22]. Moreover, pregnant women can vertically transmit ACAM2000 to the fetus which may result in fetal death [22]. Other risks associated with the ACAM2000 vaccine reported by Rizk et al. include myopericarditis and post-vaccine encephalitis.

### 3.5. Epidemiology

Since its discovery in 1958 and the first human case in 1971 [16], monkeypox has largely affected West and Central African countries and has two distinct genetic clades originating from these regions [4,16]. Whereas the Central African clade of monkeypox was found to have a fatality rate of approximately 10.6% [4] and had primarily affected children [4,7], the Western African clade had a lower observed fatality rate of 3.6% and predominantly occurred among adult populations [7]. In addition, Bunge et al. reported that monkeypox has historically affected the Democratic Republic of Congo (DRC) the most, with it being the only country where new cases have occurred annually over the past 50 years [4,7]. They also found that the median age of onset has evolved from 4–5 years to 10–21 years over the past two decades, and the virus has disproportionately affected males since its initial detection [4].

A recent review of a previous monkeypox outbreak that occurred between 2017–2018 in Nigeria showed that people infected with monkeypox were aged between 2 days and 50 years (median 29 years). About 69% of these cases were males with a 6% case fatality rate [7]. This outbreak of the West African clade of monkeypox occurred across 15 Nigerian states and the Federal Capital Territory [12]. A previous study reported that this outbreak is the largest ever recorded in Nigeria, with 276 suspected cases, including 118 confirmed, 4 probable, and 7 fatalities [7]. Another epidemiological report confirmed 4 cases originating in Nigeria in individuals travelling to the UK (*n* = 2), Israel (*n* = 1), and Singapore (*n* = 1) during this time [8]. Subsequently, one instance of human-to-human nosocomial transmission between an infected individual and a healthcare worker and two instances of transmission to a family member occurred in the UK [4]. According to Mauldin et al.’s study, no genetic or epidemiological link was found between the two individuals travelling to the UK [4,8]. It should be also noted that monkeypox cases in Nigeria were not only reported in expected rural ecological regions (e.g., mangrove swamp areas), but also were reported in dryer vegetative zones (e.g., savannahs) and in urban areas of Nigeria [4,8]. Similarly, other authors cited multiple separate instances of zoonotic transmission as the origin of the outbreak [13].

Literature on current monkeypox outbreaks remains limited and largely consists of reports from both governmental and non-governmental health organizations. From January 1st to 4 July 2022, the WHO reported 6027 laboratory confirmed cases of monkeypox across 59 of its member states [28]. This represented a 77% increase in cases occurring from June 22nd to July 4th. According to a report published on July 6th, monkeypox cases began to significantly increase in May 2022, and were clustered in the UK (*n* = 1235) and European countries, such as Germany (*n* = 1054), Spain (*n* = 802), and France (*n*= 498) [28]. Now, as of October 20, 75,166 confirmed cases of monkeypox across 109 countries have been confirmed [10]. Outside of Europe, monkeypox outbreaks are also occurring in Africa, the Americas, the Eastern Mediterranean region, and the Western Pacific region [9]. As of November 2022, Brazil (*n* = 9655), Colombia (*n* = 3630), and Peru (*n* = 3359) have the highest observed case numbers in South America [10], while Israel (*n* = 262) displays the highest case numbers in Asia [10,29]. PCR testing has confirmed that current cases in non-endemic countries belong to the West African genetic clade [9], although the ECDC has determined that first reports of transmission in Europe were not epidemiologically linked to endemic areas of West Africa [30]. 25 deaths have occurred and the WHO has updated its classification of monkeypox from a moderate global threat to a Public Health Emergency of International Concern [11]. A recent report published collaboratively by an international group of physicians found that the most common observed symptoms of monkeypox were a vesiculopustular rash (95%), lymphadenopathy (56%), fever (62%), lethargy (41%), and headache (27%) [31]. Similar symptoms were also noted by other studies conducted in Mexico [32], Spain [33] and the UK [34]. The ECDC has noted that although cases of monkeypox have been reported as mild and self-limiting, there are currently not enough cases to accurately predict future morbidity and mortality [30].

Reports published by the WHO and the ECDC confirmed that current monkeypox outbreaks are disproportionately affecting men (99%) [9] with 60% of men who reported their sexual orientation identifying as gay, bisexual, or men who have sex with men [28]. It is currently unknown how the virus is being spread through sexual contact and if bodily fluids transmitted during sex are primarily responsible [9] Moreover, atypical clinical features of monkeypox have been reported, including fewer vesiculopustular lesions overall, lesions limited only to the genital and perianal areas, and lesions occurring before the onset of fever and lymphadenopathy [9]. The WHO and ECDC have identified an elevated risk of contracting monkeypox, specifically among MSM reporting new or multiple sexual partners, as well as healthcare workers and laboratory personnel who come into direct contact with the virus without donning personal protective equipment (PPE) [9,30].

## 4. Conclusions

Although monkeypox has largely been contained to its endemic regions of West and Central Africa, much attention should be paid to frequently eradicate such rare infectious diseases. The global emergence of monkeypox has presented new challenges for public health and has called for further investigation into its epidemiological profile across international contexts. More specifically, further research on the transmission dynamics of monkeypox is needed. Researchers could explore in more depth the specific animal reservoirs implicated in the zoonotic transmission of monkeypox to inform and prevent future transmission from animals to humans. Further investigation into the sexual transmission of monkeypox is required as well as the role those bodily fluids exchanged during sex, such as semen and vaginal fluids, play in transmission need to be investigated [35]. Regarding public health response, public, private, and international health agencies should continue to collaborate with local governments to ensure that the appropriate resources supporting the surveillance, testing, outbreak response, and management of monkeypox continue to be mobilized. Resources should be made available particularly to low- and middle-income countries (LMIC) where the burden of disease may be greater due to compromised healthcare systems.
